# Jatrorrhizine Hydrochloride Suppresses Proliferation, Migration, and Secretion of Synoviocytes In Vitro and Ameliorates Rat Models of Rheumatoid Arthritis In Vivo

**DOI:** 10.3390/ijms19051514

**Published:** 2018-05-18

**Authors:** Haiwen Qiu, Shengnan Sun, Xuemei Ma, Congcong Cui, Gang Chen, Zhenzhou Liu, Hui Li, Mei Liu

**Affiliations:** Jiangsu Key Laboratory for Molecular and Medical Biotechnology and College of Life Sciences, Nanjing Normal University, Nanjing 210023, China; 15895937858@163.com (H.Q.); alyssia_s@163.com (S.S.); njnumaxuemei@163.com (X.M.); cuicongcong2011@163.com (C.C.); m18852005775@163.com (G.C.); ysszlzz@163.com (Z.L.); 15050527874@163.com (H.L.)

**Keywords:** jatrorrhizine hydrochloride, rheumatoid arthritis, collagen-induced arthritis, MH7A cells

## Abstract

Jatrorrhizine hydrochloride (JH), an active component isolated from the traditional Chinese herb *Coptis chinensis*, has been reported to have antimicrobial, antitumor, antihypercholesterolemic, and neuroprotective activities. However, its antirheumatoid arthritis (RA) property remains unknown. In this study, a collagen-induced arthritis (CIA) rat model was used to evaluate the therapeutic effects of JH on RA by using arthritis score, radiological evaluation, and histopathological assessment. The in vitro effects of JH on proliferation, migration, and production of inflammatory mediators in RA-derived fibroblast-like synoviocyte MH7A cells were determined by the EdU incorporation assay, wound healing assay, real-time PCR, and ELISA, respectively. The in vivo studies showed that JH treatment significantly prevented the progression and development of RA in CIA rats through anti-inflammation and suppressing bone destruction. The in vitro studies revealed that JH could effectively attenuate the destructive phenotypes of MH7A cells, including inhibiting proliferation, migration, and production of inflammatory mediators. Further mechanistic analysis demonstrated that JH suppressed tumor necrosis factor alpha (TNFα)-stimulated activations of nuclear factor of kappaB (NF-κB) and mitogen-activated protein kinases (MAPKs) (ERK and p38) leading to the downregulation of proinflammatory cytokines, which might be beneficial to the antiproliferative and antimigratory activities of FLS cells. Collectively, our results demonstrated that JH has a great potential to be developed into a novel therapeutic agent for treating RA.

## 1. Introduction

Rheumatoid arthritis (RA) is a systemic autoimmune disease characterized by joint inflammation and bone erosions [1]. Despite the etiology and pathogenesis of RA being poorly understood, several cell types including T cells, macrophages, B cells, osteoclasts, and chondrocytes, have been demonstrated to be involved in destructive processes of the RA joint [2,3,4]. Recently, increasing evidence indicates that activated RA fibroblast-like synoviocytes (FLSs), which are present in great numbers in rheumatoid arthritis synovium, exhibit the characteristics of malignant cells and participate in almost all of the pathological events [5,6,7]. The aggressive proliferation and migration of FLSs and their secretions such as proinflammatory cytokines and matrix-degrading enzymes lead to synovial hyperplasia, persistent synovitis, and joint damage in RA patients [6,8]. Furthermore, the activated and destructive properties of FLSs have been demonstrated to tightly correlate with radiographic and histological damage in RA and its rodent models [9,10]. Hence, the exploration of new antirheumatic drugs to attenuate the destructive behaviors of RA–FLS could be a reasonably efficient way for treating RA.

Many current clinical drugs for RA treatment such as disease-modifying antirheumatic drugs (DMARDs), glucocorticoids, and biological agents have been proven to relieve the severity of RA, slow this disease progression, and prevent the subsequent joint damage [11,12]. However, the use of these therapies has been limited because of their obvious adverse effects with a high frequency and high cost of treatment [13,14,15]. Therefore, searching for novel potential antiarthritic drugs especially from natural products is becoming an area of active research.

Jatrorrhizine hydrochloride (JH), one of the major protoberberine alkaloids isolated from many medicinal plants, such as *Berberis aristata* and *Coptis chinensis*, has been demonstrated to possess antimicrobial, antioxidant, hypoglycemic, and neuroprotective effects [16,17,18,19]. However, its effect on RA has not been elucidated up to now. Notably, according to the structural comparison, jatrorrhizine showed a very similar structure to another alkaloid in *Coptis chinensis*, berberine, both of which belong to a kind of quaternary protoberberine alkaloid. The previous study has proved that berberine exhibited antiarthritic activity through inhibiting inflammation and neovasculature development [20]. Thus, we speculated that jatrorrhizine hydrochloride may have a potential role of antirheumatoid arthritis. Type II collagen-induced arthritis (CIA) model has been widely used to evaluate new antiarthritic drugs, as it shares clinical, histological, and immunological features with RA [21]. In the present study, we investigated the therapeutic effect of JH on CIA in rats, and subsequently investigated the possible protective mechanisms.

## 2. Results

### 2.1. JH Treatment Suppressed Synovial Inflammation and Joint Damage 

**Figure 1:**
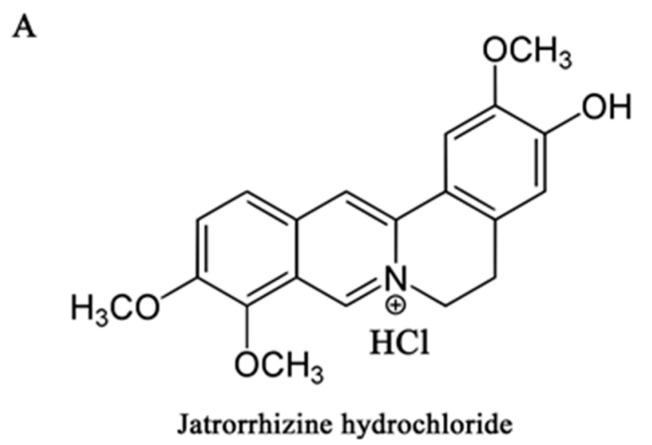

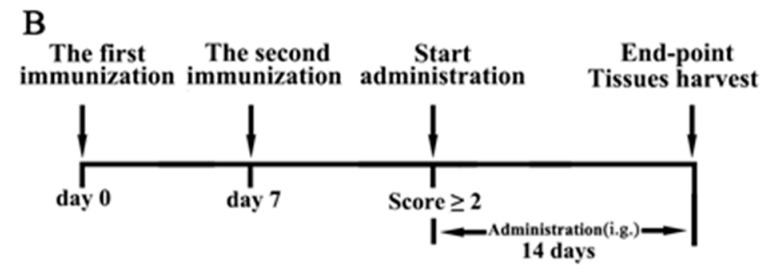
(**A**) The chemical structure of jatrorrhizine hydrochloride (JH); (**B**) The timeline diagram of the process of CIA introduction and treatment. Female Wistar rats were immunized on day 0 and 7, respectively. When arthritis was established (clinical score ≥ 2), the CIA rats were randomly grouped and intragastrically administrated with different concentrations of JH (low dose, 20 mg·kg^−1^·day^−1^; high dose, 50 mg·kg^−1^·day^−1^) or vehicle (0.9% saline) every day for a 14-day period. The MTX-treated group was intragastrically treated with MTX (3 mg·kg^−1^ body weight) every 3 days as a positive agent. At the endpoint of the experiment, rats were killed and tissues were collected for further detection.

**Figure 2:**
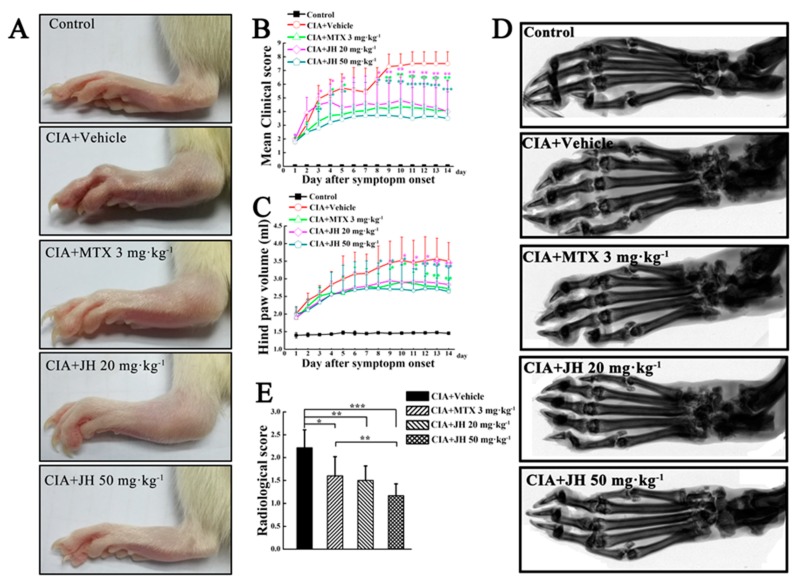
JH treatment significantly suppresses synovial inflammation and joint damage. (**A**) Representative photographs of the hind paws of CIA rats at day 14 after symptom onset; Clinical score (**B**) and paw swelling (**C**) were inhibited by JH or MTX. * *p* < 0.05, ** *p* < 0.01 and *** *p* < 0.001 versus vehicle-treated CIA rats; (**D**) Representative radiographs of the hind paws of CIA rats at day 14 after symptom onset; (**E**) Radiological scores were analyzed as described in Materials and Methods section. The values shown are the mean ± SD. *n* = 6 in age-matched group and *n* = 7 in vehicle and MTX- or JH-treated groups, respectively. * *p* < 0.05, ** *p* < 0.01 and *** *p* < 0.001.

To investigate the effect of JH on the development of CIA rats after disease onset (clinical score ≥ 2), JH with different dosages (20 mg∙kg^−1^ and 50 mg∙kg^−1^) was intragastrically given every day for a 14-day period (Figure 1A,B). As shown in Figure 2A,B, macroscopic evidence of arthritis such as erythema or swelling was markedly observed in vehicle-treated CIA rats, while JH treatment significantly blocked the development and progression in CIA rats. Consistent with the clinical scoring, measurements of paw swelling in CIA rats also showed JH to be highly effective (Figure 2C). Compared to 3 mg∙kg^−1^ of methotrexate (MTX) which markedly attenuated arthritis severity in CIA rats, JH treatment with 50 mg∙kg^−1^ showed a stronger effect as assessed by inflammatory scores and paw swelling, demonstrating a dramatic therapeutic potential in CIA animal model (Figure 2A–C).

Next, we used radiography to investigate the effect of JH on articular destruction in CIA rats. Under X-ray imaging conditions, the vehicle-treated CIA rats showed changes typical of RA, with obviously observed bone erosions, articular destruction, joint space narrowing, and joint displacement (Figure 2D). However, the bone destruction was significantly attenuated in groups receiving 20 mg∙kg^−1^ and 50 mg∙kg^−1^ of JH or MTX (Figure 2D,E), suggesting JH could exert antibone destructive role in addition to its anti-inflammation presented in the clinic score and paw thickness data. 

As a preliminary study to assess the potential side effects of JH in CIA rats, the body and organ weights, and serum alanine aminotransferase (ALT) and aspartate aminotransferase (AST) levels were measured. Compared with the control rats, the vehicle-treated CIA rats showed a body weight loss (Table 1). However, JH significantly suppressed the loss of body weight of CIA rats. In addition, JH-treated CIA rats did not show any significant changes in organ weights, as well as ALT and AST levels, as compared with the untreated CIA rats and control rats, suggesting that the present JH treatment probably had no significant adverse side effects.

**Figure 3:**
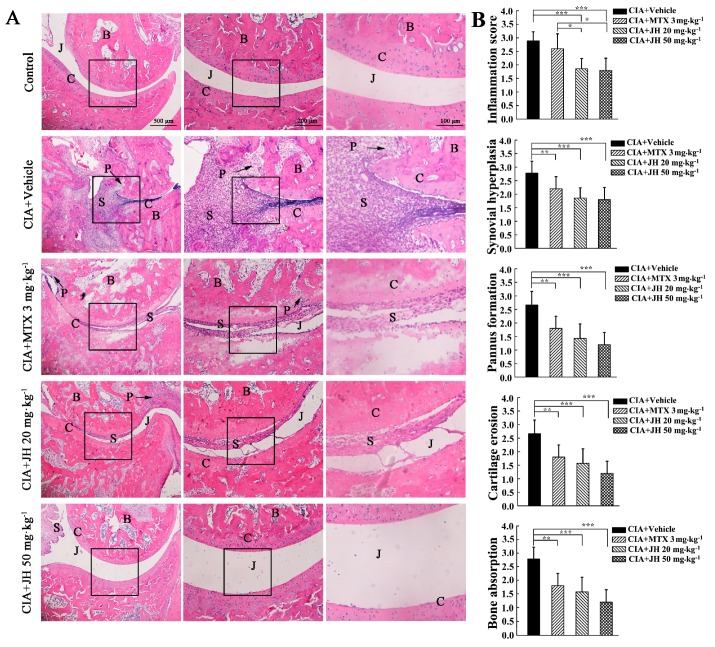
Effect of JH on pathologic changes of rats with CIA. (**A**) Representative hematoxylin and eosin-stained sections of rat ankles from different groups. Middle panel: enlarged image in the black-square zone in the left panel; Right panel: enlarged image in the black-square zone in the middle panel; (**B**) Histologic scores were determined as described in Materials and Methods section. B, bone; C, cartilage; J, joint space; P, pannus; S, synovium. Data are presented as means ± SD (*n* = 6–7 per group). * *p* < 0.05, ** *p* < 0.01 and *** *p* < 0.001.

To further assess the effect of JH on developed arthritis, a histopathological evaluation was performed by semiquantitative grading scales. The results suggested the pathological features of RA were obviously observed in the ankle joints of vehicle-treated CIA rats including inflammatory cell infiltration, synovial hyperplasia, pannus formation, cartilage destruction, and bone erosion. In contrast, JH or MTX significantly attenuated the above-described structural changes (Figure 3A,B), further demonstrating the protective effect of JH on RA.

### 2.2. JH Treatment Significantly Suppressed the Production of Anti-CII Antibody and Proinflammatory Cytokines in CIA Rats

**Figure 4:**
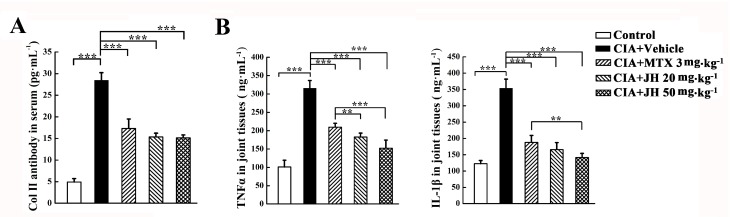
JH treatment significantly suppresses the production of anti-CII antibody and proinflammatory cytokines in CIA rats. (**A**) Serum anti-CII antibody was detected by ELISA, as described in Material and Methods section; (**B**) TNFα and IL-1β in joint homogenates were determined with the use of commercial kits according to the manufacturer’s instructions. Data are presented as means ± SD (*n* = 6–7 per group). ** *p* < 0.01 and *** *p* < 0.001.

We next investigated the mechanisms underlying the decreased severity of CIA following JH treatment. It has been reported that anti-CII antibody is involved in the development of arthritis in CII-immunized animals [22]. In order to investigate whether JH affects anti-CII antibody production, we measured the concentrations of anti-CII antibody in the sera of the rats at the end of treatment. As shown in Figure 4A, the level of anti-CII antibody was significantly elevated in CIA rats as compared to control rats. However, this elevation was significantly inhibited by JH treatment, which was comparable to that of MTX-treated rats. The inhibitory effect of JH on autoantibody production maybe contribute, at least in part, to its antiarthritic action. 

The previous studies demonstrated that proinflammatory cytokines are involved in joint inflammation and arthritis progression [23,24,25]. Therefore, we collected the joint tissues from the CIA rats to measure the levels of TNFα and IL-1β by ELISA. Compared with vehicle-treated CIA rats, significant decreases in TNFα and IL-1β were observed in the joint tissues of MTX- or JH-treated rats (Figure 4B), suggesting JH might provide beneficial effects by downregulating the synthesis of inflammatory cytokines. 

### 2.3. JH Decreased TNFα-Stimulated Proliferation and Migration in Cultured MH7A Cells

**Figure 5:**
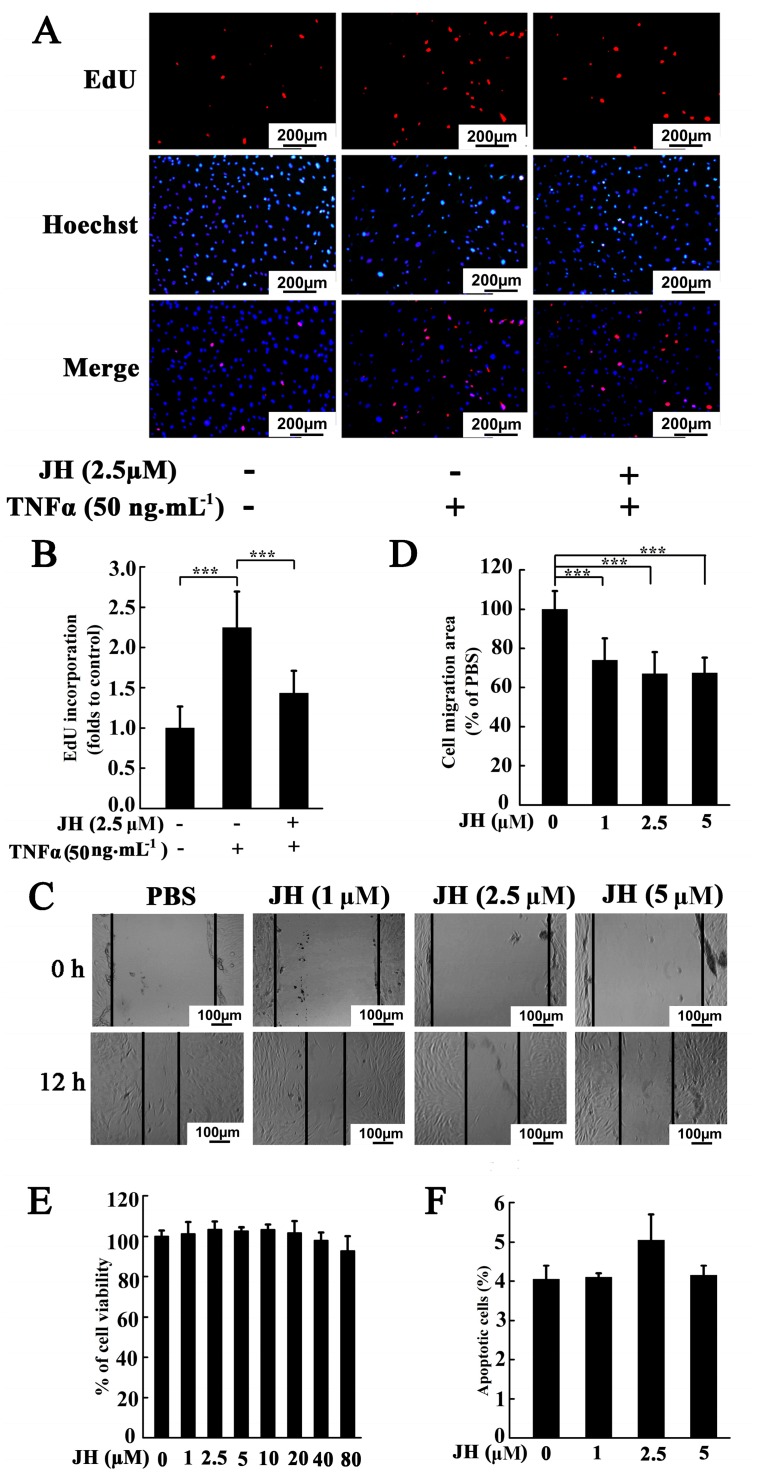
JH reduces TNFα-induced proliferation and migration of MH7A cells. (**A**,**B**) EdU incorporation assay showed that JH (2.5 µM) significantly inhibited the TNFα-induced proliferation of MH7A cells. Data are presented as means ± SD of three independent experiments (*** *p* < 0.001); (**C**,**D**) Wound healing assay showed that JH inhibited the TNFα-induced migration of MH7A cells. Serum-starved cells were pretreated with JH for 1 h and then stimulated with TNFα for 12 h. Cell migration was then measured. Data are presented as means ± SD of three independent experiments (*** *p* < 0.001); (**E**) Effect of JH on MH7A viability was measured by MTS; (**F**) The percentage of apoptotic cells within each population was determined by FACS, as described in Materials and Methods section. Data are represented as mean ± SD of three independently prepared samples.

To investigate whether the therapeutic effect of JH was because of an attenuation of the destructive behaviors of rheumatoid FLS, we cultured human synovial fibroblast cell line MH7A and tested JH-treated proliferation, migration, and apoptosis. As shown in Figure 5A,B, TNFα stimulation could dramatically increase the cell proliferative potential, however, this increase was significantly inhibited by 2.5 µm JH. We next used a wound-healing assay to determine the effect of JH on the migration of MH7A. As shown in Figure 5C,D, JH significantly reduced cell migration area at doses greater than 1 μM. In order to exclude the potential cytotoxic effects of JH in MH7A cells, a cell viability assay was performed. As shown in Figure 5E, JH did not inhibit cell viability, even at a concentration as high as 80 μM. To investigate the apoptosis-inducing effect of JH, the apoptotic rate of JH-treated MH7A cells was detected by flow cytometry. The results showed that JH did not affect apoptosis at the given dosages (Figure 5F), indicating that the inhibitory action of JH on proliferation and migration of MH7A was not due to apoptosis.

### 2.4. JH Treatment Inhibited TNFα-Stimulated Inflammatory Cytokine Production in Cultured MH7A Cells

**Figure 6:**
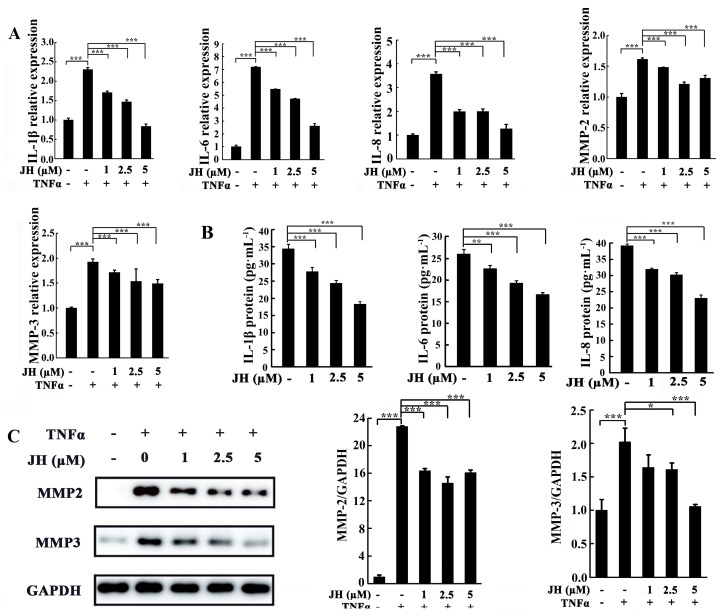
JH significantly suppresses the production of cytokines in TNFα-induced MH7A cells. MH7A cells were pretreated with different doses of JH for 1 h and then stimulated with TNFα (50 ng·mL^−1^) for 24 h. (**A**) The transcript levels of cytokines were determined by real-time PCR including *IL-1β*, *IL-6*, *IL-8*, *MMP-2*, and *MMP-3*. Expression of each target gene was calculated as a relative expression to *β-actin* and represented as a fold change over the TNFα-untreated, JH-untreated cells. Data are presented as means ± SD of three independent experiments (*** *p* < 0.001); (**B**) The culture supernatants were collected, and ELISA was used to examine the protein expression of IL-1β, IL-6 and IL-8. *n* = 3, ** *p* < 0.01 and *** *p* < 0.001; (**C**) The cells were lysed and Western blot was performed to measure the protein product of MMP-2 and MMP-3. Also, the ratio of the density of MMP-2 and MMP-3 relative to GAPDH band was determined using Image J, respectively. *n* = 3, * *p* < 0.05 and *** *p* < 0.001.

JH has been demonstrated to inhibit inflammatory response in CIA rats. To further examine the anti-inflammatory role of JH in RA, MH7A cells were treated with various concentrations of JH and subsequently stimulated with TNFα. As shown in Figure 6A, TNFα stimulation increased the transcript levels of cytokines including *IL-1β*, *IL-6*, *IL-8*, *MMP-2*, and *MMP-3*. However, these increases were significantly inhibited by JH treatment. In consistence with the real-time PCR data, the protein levels of IL-1β, IL-6, IL-8, MMP-2, and MMP-3 were also demonstrated to be reduced by JH (Figure 6B,C), which further confirmed the anti-inflammatory effect of JH on RA.

### 2.5. JH Treatment Inhibited TNFα-Stimulated Activations of MAPKs and NF-κB 

**Figure 7:**
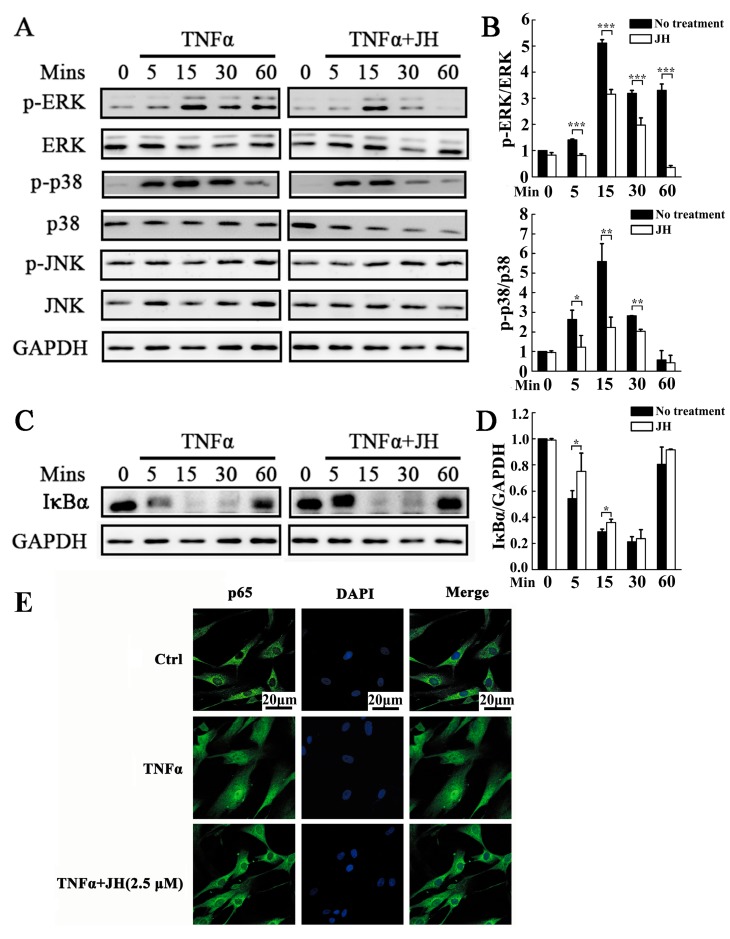
JH inhibits TNFα-induced activations of MAPK (ERK and p38) and NF-κB in MH7A cells. (**A**,**C**) MH7A cells were pretreated with JH (2.5 µM) for 1 h, then induced with TNFα (50 ng·mL^−1^) for 0, 5, 15, 30, and 60 min. Protein was then extracted for Western blot analyses using antibodies against p-ERK1/2, total ERK1/2, p-p38, total p38, p-JNK, total JNK, IκBα and GAPDH; (**B**,**D**) Relative expressions were determined by densitometric analysis. The ratios of the density of IκBα bands relative to GAPDH bands and each phosphorylated MAPK band relative to its total protein counterpart were determined using Image J. Data are presented as means ± SD of three independent experiments (* *p* < 0.05, ** *p* < 0.01 and *** *p* < 0.001); (**E**) MH7A cells were treated with JH (2.5 µM) for 4 h, and then stimulated with TNFα (50 ng·mL^−1^) for 30 min. The localization of p65 was visualized by immunofluorescence analysis.

To explore the mechanisms through which JH attenuated the destructive behaviors of MH7A and further prevented the progression of RA, TNFα-induced MAPK and NF-κB signaling pathways were investigated. As shown in Figure 7A,B, upon stimulation with TNFα, three MAPK family members, extracellular signal-regulated kinase (ERK), c-Jun N-terminal kinase (JNK), and p38 kinase, showed increased phosphorylation. By comparison, cotreatment of MH7A cells with TNFα and JH significantly decreased phosphorylation of ERK and p38. However, JH had no effect on TNFα-stimulated JNK activation (Figure 7A). In addition to MAPKs signaling pathways, we also tested the activation of NF-κB, which has been demonstrated to play a pivotal role in the development and progression of RA. As shown in Figure 7C,D, TNFα-stimulated MH7A cells exhibited robust NF-κB activation as evidenced by enhanced IκBα degradation. This enhancement was inhibited in JH-treated cells. It is well known that in unstimulated cells, NF-κB subunits are retained in the cytoplasm by binding to the inhibitory IκB protein. Phosphorylation and subsequent degradation of IκBα liberates NF-κB proteins (such as p65) to enter the nucleus and trigger downstream gene expression [26]. In this study, we observed that NF-κB p65 was translocated into nuclei following TNFα stimulation. However, JH substantially suppressed p65 nuclear translocation, as demonstrated by the retention in the cytoplasm of the p65 proteins.

## 3. Discussion

JH has been reported to exhibit multiple pharmacological and biological properties, including antioxidation [27], bacteriostasis [16,28], tumor cell growth suppression [29,30], antidiabetic, and antihyperlipidemic activities [31,32]. Nevertheless, to the best of our knowledge, no study has investigated its antiarthritic effect on animal model or humans. In this study, we demonstrated, for the first time, that JH could effectively suppress the development and progress of RA by inhibiting inflammatory response and joint destruction in CIA rats. These beneficial effects may occur via attenuating the destructive behaviors of rheumatoid FLS.

It is well known that the most significant characteristic of RA is the intensive inflammation that is out of control [33]. Managing to control the inflammation could prevent the disease progression, which would be the optimal strategy for RA therapy. In the present study, we demonstrated that JH effectively attenuated the severity of RA though suppressing inflammatory responses. First of all, JH inhibited local levels of TNFα and IL-1β in CIA rats. Further, JH decreased the production of proinflammatory cytokines including IL-1β, IL-6, and IL-8 in cultured MH7A cells. Finally, JH suppressed inflammatory responses in CIA rats at the clinical and histopathological levels. A cascade of inflammatory cytokines have been demonstrated to contribute to the vicious circle of synovial inflammation [34], therefore, we speculated that the beneficial anti-inflammatory effect of JH in RA might be attributed to its ability, at least in part, to suppress the synthesis of a serial of inflammatory cytokines.

Destruction of bone is the hallmark of RA, and controlling these erosive processes is the most challenging objective in the treatment of RA. In this study, we found that JH not only protected against joint inflammation but also suppressed joint destruction in CIA animal model. Our radiographic and histologic analyses revealed that subchondral bone resorption, a characteristic feature of CIA, was mitigated by JH. Since inflammation is well known to trigger osteoclastogenesis and bears the primary responsibility for bone resorption in RA, we cannot exclude the possibility that JH prevented bone destruction through the indirect effect of anti-inflammation. However, we demonstrated, in another unpublished manuscript, that JH could directly target osteoclast differentiation and function (unpublished data). Therefore, the antibone destructive effect of JH might attribute to a direct enabling effect on osteoclast formation, or a synergistic effect of anti-inflammation and antiosteclastogenesis. Further studies are warranted to investigate the antibone destructive actions of JH in RA.

FLSs have been demonstrated to participate in almost all of the pathological events and play an important role in RA pathogenesis [5,35]. The aggressive proliferation of FLS and their secretions such as proinflammatory cytokines and matrix-degrading enzymes lead to synovial hyperplasia, persistent synovitis, and joint damage in RA patients [6]. In our study, JH was found to attenuate the destructive behaviors of RA-derived fibroblast-like synoviocyte MH7A cells through inhibiting proliferation, migration, and secretion of cytokines and MMPs including IL-1β, IL-6, IL-8, MMP-2, and MMP-3. The EdU incorporation assay showed that at the dose of 2.5 μM, JH markedly decreased the proportion of EdU-positive cells, which was further demonstrated by the in vivo data. We found that JH significantly attenuated the synovial hyperplasia in CIA rats. The wound healing assay revealed that JH was able to reduce the migration rate of MH7A cells. This decreased migration was further confirmed by the suppressed pannus formation in JH-treated CIA rats in vivo. In addition to the suppressed expressions of proinflammatory cytokines, the mRNA and protein levels of MMP-2 and MMP-3 were also reduced by JH. It is well known that MMPs, mainly produced by FLS in RA, are proteases that participate in the remodeling of the extracellular matrix and play important roles in FLS migration and the progressive destruction of joints in RA [36,37]. MMP-2 was reported to be capable of cleaving gelatine, type I, IV, and V collagens, elastin, and vitronectin, and provided condition for the cell migration and invasion [38]. MMP-3 was demonstrated to be expressed higher in SF than in the systemic circulation [39] and it was proposed as an important indicator of radiological progression in early RA [40]. Similar to these previous findings, JH was found to significantly decrease the production of MMP-2 and MMP-3 in MH7A cells, further demonstrating the antimigratory and antibone destructive properties of JH in disease control.

**Figure 8:**
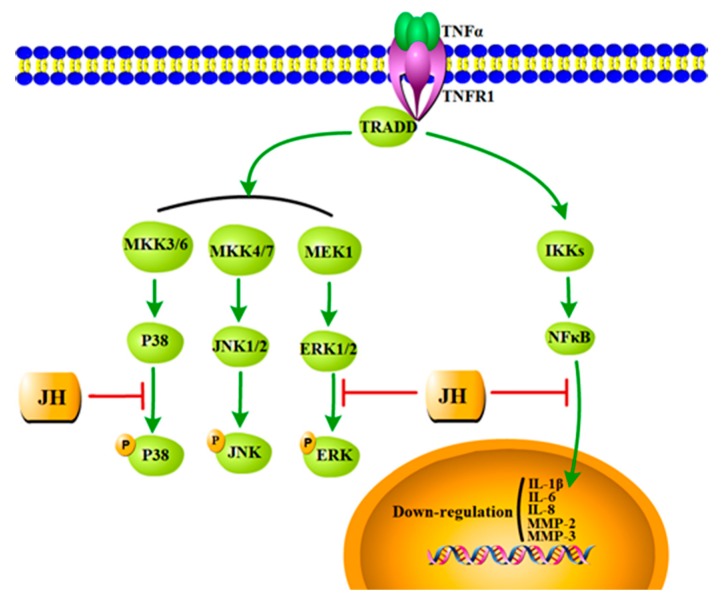
Schematic diagram of the mechanism by which JH attenuated the destructive behaviors of MH7A and further prevented the progression of CIA. In TNFα stimulated MH7A cells, MAPK and NF-κB signaling pathways were activated. JH treatment significantly decreased phosphorylation of ERK and p38. Also, NF-κB activation was inhibited through modulating IκBα protein degradation and thereby suppressing the nuclear translocation of the p65 subunit of NF-κB. Accordingly, NF-κB target genes including *IL-1β*, *IL-6*, *IL-8*, *MMP-2* and *MMP-3* were downregulated.

Having established that JH plays an inhibitory role in CIA model and cultured MH7A cells, we further analyzed the possibly involved signaling mechanisms. We found that the decreased activations of NF-κB and MAPKs might account for the beneficial effects of JH in RA (Figure 8). MAPKs have been demonstrated to regulate an array of key cellular processes including cell survival/apoptosis, proliferation, and differentiation as well as inflammatory responses [41,42]. In activated rheumatoid FLS, MAPKs have been reported to be universally upregulated [43]. The reversion of these changes usually is deemed as a therapeutic aim to control inflammatory responses. In this regard, we examined the effects of JH on TNFα-induced activation of MAPKs in MH7A cells. The Western blot results showed that JH inhibited phosphorylations of ERK and p38, but without any effect on p-JNK. These results suggest that the inhibitory effect of JH on the production of inflammatory mediators could occur through blocking of the ERK and p38 signaling pathway. And this decrease in proinflammatory cytokines levels might lead to the suppressed proliferative activity on RA–FLS. In addition to MAPKs signaling pathways, NF-κB activation has also been demonstrated to contribute to the pathogenesis of RA by activating the transcription of inflammatory cytokines and MMPs [44,45]. Suppression of the NF-κB pathway could be considered a novel strategy for delaying the progress of RA. In this study, we demonstrated an inhibitory effect of JH on NF-κB activation through modulating IκBa protein degradation and thereby suppressing the nuclear translocation of the p65 subunit of NF-κB. This was further confirmed by the finding that JH significantly decreased the expressions of a subset of NF-κB target genes including *IL-1β*, *IL-6*, *IL-8*, *MMP-2*, and *MMP-3*. Taken together, our data revealed that JH could exert a therapeutic effect on RA via multiple targets (Figure 8), and further investigations are required to unveil its direct binding sites.

It is a limitation in our study that the in vitro experiments were performed using the immortalized MH7A cell line but not primary rheumatoid FLS. It is well known that primary cells can better parallel actual activity of FLS in RA patients. However, primary cells pose several problems that constrain their usefulness, i.e., the limited source, low growth potential, and lot-to-lot variability in functional assays. Thus, MH7A has become an important alternative model for understanding the pathogenesis of RA and evaluating new antiarthritic drugs. Another limitation is that except for synoviocytes, we did not detect the effect of JH on the other cells. As is known, synovial inflammation is driven by a complex interaction between activated synoviocytes and infiltrating cells such as T cells, B cells, macrophage, and neutrophil. These cells drive each other’s activation and survival by paracrine production of cytokines. Since it is the FLS that form the aggressive pannus and are the major effectors of tissue damage through production of extracellular matrix degrading enzymes, our study focused on the inhibitory effect of JH on the behaviors of MH7A cells. The modulation of JH on the other cells and their interactions are need for further investigation. 

Throughout the present study, JH was well-tolerated at the given doses with no evidence of drug toxicity and no damage to liver function in the treated rats. Additionally, the in vitro study showed that JH had no cytotoxic effect on rheumatoid FLS cells. Therefore, it is a promising complementary or alternative medicine for RA therapy because of its effectiveness and safety. 

## 4. Materials and Methods

### 4.1. Animals

A total of 34 female Wistar rats (160–180 g, 6–7 weeks old) were purchased from Shanghai Slac Laboratory Animal Co Ltd. (Shanghai, China). They were housed under specific pathogen-free (SPF) conditions (22 °C, 12 h/12 h light/dark, 50–55% humidity) and given free access to food and water. All the animal experiments were approved by the Experimental Committee of Nanjing Normal University (#20160101, approved date: 22 December 2015).

### 4.2. Reagents

Jatrorrhizine hydrochloride (Purity ≥ 98%) was purchased from Nanjing Zelang Medical Technology Co. Ltd (Nanjing, China) (Figure 1A). MTX was obtained from Shanghai Sine Pharmaceutical Co., Ltd. (Shanghai, China). Dulbecco’s modified Eagle’s medium (DMEM) and fetal bovine serum (FBS) were purchased from Gibco (Gibco BRL, Grand Island, NY, USA). Recombinant tumor necrosis factor alpha (TNFα) was purchased from Peprotech (PeproTech, Inc., Rocky Hill, NJ, USA). MTS reagents were obtained from Sigma-Aldrich (St. Louis, MI, USA). Trizol reagent was obtained from Invitrogen (Carlsbad, CA, USA). Primary antibodies targeting phosphorylated (p) ERK, p-p38, p-JNK, ERK, p38, JNK, IκBα, and GAPDH were obtained from Santa Cruz Biotechnology (Dallas, CA, USA). Anti-p65, anti-MMP2, and anti-MMP3 antibodies were purchased from CST (Cell Signaling Technology Inc., Beverly, MA, USA). Commercial kits for ALT and AST measurement were from Jiancheng Institute of Biotechnology (Nanjing, China). TNFα and IL-1β ELISA kits were purchased from CUSABIO BIOTECH CO., Ltd (Wuhan, China). Lyophilized native chicken type II collagen (CII) was purchased from Sigma Chemical Company (St. Louis, MO, USA).

### 4.3. Induction of CIA and Treatment with JH

Female Wistar rats were immunized intradermally with 1.5 mg native chicken collagen type II with an equal volume of Freund’s complete adjuvant (Sigma, St. Louis, MO, USA). Seven days later, the rats were then given a subcutaneous booster injection of half the amount of CII emulsified in Freund’s incomplete adjuvant. The control rats were injected in the same way with saline (*n* = 6). All immunized rats developed CIA (clinical score ≥2) after a mean (± SD) interval of 15 ± 1 days. Rats with CIA were then randomly divided into four groups as model group, MTX-treated group, and two JH-treated groups (*n* = 7 per group). JH-treated groups were intragastrically administrated with different doses of JH (20 and 50 mg∙kg^−1^ body weight, respectively) and the model group was treated with vehicle (0.9% saline) every day for a 14-day period. The doses of JH were determined according to previous studies [31,32] with modification from our preliminary experiments. The MTX-treated group received intragastrical administration with MTX (3 mg·kg^−1^ body weight) every 3 days (according to clinical usage) as a positive agent. A timeline process of CIA introduction and treatment is shown in Figure 1B. Since day 9 after the first immunization, clinical arthritis scores were evaluated daily using a scoring system of 0–4 for each limb: 0 = no arthritis; 1 = swelling and/or redness of one to two interphalangeal (IP) joints; 2 = involvement of three to four IP joints or one larger joint; 3 = more than four joints red/swollen; 4 = severe arthritis of an entire paw, yielding a score between 0 and a maximum of 16 per animal. In addition, the paw swelling was measured by a volume displacement plethysmometer (YLS-7A, Facility Station of Shandong Academy of Medical Science, Shandong, China). Scoring and paw volume measurements were carried out by two independent observers. After a 14-day treatment, serum was collected for measurement of anti-CII antibody, ALT, and AST. Joint tissues were harvested for assessment of radiography, histology, and proinflammatory factor secretion. In addition, the change in body weight (%) of each individual CIA rat after the onset of arthritis was calculated as previously reported [46]. The formula is as follows: change in body weight = [(body weight of day 14 arthritis/body weight of day 1 arthritis) − 1] × 100%.

### 4.4. Radiographic Evaluation

The left hind paws of rats were exposed under X-ray using Faxitron X-ray Corporation MX-20. The radiological analysis was evaluated with a scoring system according to the previous report [47]. In brief, the degrees of bone erosion were scored on a scale of 0–3: 0 = normal, 1 = mild changes, 2 = moderate changes, 3 = severe changes. The radiological evaluation was scored by independent observers in a blinded manner.

### 4.5. Histopathological Assessment

After radiographic evaluations, the ankle joints were fixed in 4% paraformaldehyde (Sigma-Aldrich, St. Louis, MI, USA), decalcified in 12% disodium EDTA and embedded in paraffin. Sections (4 µm) were stained with haematoxylin and eosin (H&E) and scored for changes in inflammation, synovial proliferation, pannus formation, cartilage damage and bone erosion, each was scored on a scale of 0–4, as previously described [48]. Histopathological analysis was evaluated by independent observers in a blinded manner.

### 4.6. Serum ALT and AST Measurement

The blood was collected, centrifuged, and the supernatant was stored at −80 °C until analysis. Serum ALT and AST levels in serum were determined with the use of commercial kits according to the manufacturer’s protocols.

### 4.7. Serum Anti-CII Antibody Measurement

Serum anti-collagen type II antibody was measured by a standard ELISA kit (CUSABIO BIOTECH CO., Ltd, Wuhan, China) according to the manufacturer’s instructions. In brief, standard or serum from the CIA rats was added into the 96-well plates precoated with type II collagen. After 2 h incubation at 37 °C, the plates were washed, and then the CII-specific antibody was detected with biotin-conjugated anti-rat immunoglobulin (Ig) G antibody. The absorbance at 450 nm was measured with a microplate reader (Multiskan FC, Thermo Scientific, Shanghai, China).

### 4.8. Cytokine Levels in Rats with CIA

The right paws of rats were homogenized according to the previous report [49]. TNFα and IL-1β in joints were measured by specific ELISA kits according to the manufacturer’s instructions.

### 4.9. Cell Culture

MH7A cells were obtained from Jennio Biotech Co., Ltd. (Guangzhou, China), and cultured in DMEM supplemented with 10% FBS, 100 U∙mL^−1^ penicillin and 100 μg∙mL^−1^ streptomycin at 37 °C in 5% CO_2_ atmosphere. Cells were passaged every 3–4 days. Cells obtained from the 4th–10th passages were used in the experiment.

### 4.10. Cell Viability Assay

MTS assay was used to determine the effect of JH on the viability of MH7A. The cells were cultured overnight for attachment and then treated for 48 h with serial dilutions of JH (0–80 μM). MTS/PMS mixture was added to incubate for another 4 h according to the manufacturer’s instructions. Changes in absorbance at 490 nm were measured in a spectrophotometer (Model 680, Bio-Rad, Hercules, CA, USA). The effect of JH on cell viability was expressed as percent cell viability with vehicle treated control cells set at 100%.

### 4.11. TNFα-Induced Cell Proliferation

An EdU incorporation assay was used to analyze cell proliferation through measuring DNA synthesis. MH7A cells were pretreated with JH (2.5 μM) for 6 h and then incubated with or without TNFα (50 ng∙mL^−1^) in the medium for another 24 h. Six hours before termination of the experiments, 10 μM EdU (Sigma-Aldrich, St. Louis, MI, USA) was added to measure the incorporation of EdU into newly synthesized DNA according to the manufacture’s protocol. The cell nuclei were counterstained with Hoechst 33342. The cell proliferation rate was calculated as the proportion of nucleated cells incorporating EdU in five high-power fields per well.

### 4.12. Wound Healing Assay

A wound healing assay was used to analyze the effect of JH on MH7A migration rate. Briefly, MH7A cells were plated in 24-well plates at 70–80% confluence and serum starved overnight. A linear scratch was formed using a sterile 200 mL pipette tip (set as 0 h) and wounded monolayers were washed with PBS to remove detached cells and debris. Cells were incubated with JH or vehicle for 1 h, and then TNFα (50 ng∙mL^−1^) was added into the wells to stimulate for another 12 h. By using direct microscopic visualization, a reference point at the bottom was created in each field of the wound at 0 h, and wound closure rates were analyzed by photographing and measuring the remaining cell-free area in the identical field immediately after 12 h stimulation.

### 4.13. Analysis of Apoptosis by Flow Cytometry

Apoptosis assay was performed using Annexin V-FITC apoptosis detection kit (e-Bioscience, San Diego, CA, USA) according to the manufacturer’s protocol. Briefly, MH7A cells were exposed to JH with different concentrations (0, 1, 2.5, and 5 μM) for 24 h. Cells were then suspended in binding buffer and stained with Annexin V and propidium iodide (PI) solution. Flow cytometric analysis was performed with FACScan (Becton Dickinson, Franklin Lakes, NJ, USA) with the CellQuest program. The results were expressed as the means ± SD percentages of apoptotic cells (*n* = 3). 

### 4.14. Cytokine Levels in Cultured MH7A Cells 

To investigate the inhibitory effect of JH on inflammation, we measured the cytokines production in TNFα-induced MH7A cells at mRNA and protein levels. MH7A cells were cultured into 12-well plates overnight and then pretreated with different concentrations of JH (0, 1, 2.5, and 5 µM) for 1 h. Subsequently, TNFα (50 ng∙mL^−1^) was added to incubate for 24 h. Total RNA was extracted, and real-time PCR was used to analyze the mRNA levels of the indicated cytokines. The culture supernatants were collected, and ELISA was used to examine the protein expression of IL-1β, IL-6, and IL-8. The cells were lysed, and Western blot was performed to measure the protein product of MMP-2 and MMP-3.

For real-time PCR, total RNA was prepared using TRIzol reagent (Invitrogen Life Technologies, Carlsbad, CA, USA), and subjected to cDNA synthesis according to the manufacturer’s instructions. The following real-time PCR primers were used: *IL-1β*, 5′-ATGATGGCTTATTACAGTGGCAA-3′ (forward), 5′-GTCGGAGATTCGTAGCTGGA-3′ (reverse); *IL-6*, 5′-AACCTGAACCTTCCAAAGATGG-3′ (forward), 5’-TCTGGCTTGTTCCTCACTACT-3′ (reverse); *IL-8*, 5′-CATACTCCAAACCTTTCCACCCC-3′ (forward), 5′-TCAGCCCTCTTCAAAAACTTCTCCA-3′ (reverse); *MMP-2*, 5′-CGGTGCCCAAGAATAGATG-3′ (forward), 5′-AAAGGAGAAGAGCCTGAAGTG-3′ (reverse); *MMP-3*, 5′-CGGTTCCGCCTGTCTCAAG-3′ (forward), 5′-CGCCAAAAGTGCCTGTCTT-3’ (reverse); *β-actin*, 5′-CCACACTGTGCCCATCTACG-3′ (forward), 5′-AGGATCTTCATGAGGTAGTCAGTCAG-3′ (reverse). Real-time PCR was performed using a SYBR Premix Ex Tag kit (TaKaRa Biotechnology Co., Ltd., Dalian, China) and run in Mastercycler ep realplex 2 systems (Eppendorf, Hamburg, Germany). Each experiment was repeated three times with three parallel reactions. Expression of each target gene was calculated as a relative expression to *β-actin* and represented as fold change over the TNFα-untreated, JH-untreated cells.

For ELISA assay, the culture supernatants were harvested and centrifuged at 10,000× *g* for 5 min to remove the particulate matter. The determinations and analysis of the samples were assisted by Sino-UK Institute of Biological Technology (Beijing, China) according to the standard ELISA protocol.

For the Western blot assay, the cells were lysed and the protein was extracted. MMP-2 and MMP-3 were measured by Western blot as described below.

### 4.15. Western Blot Analysis

MH7A cells were homogenized with radioimmunoprecipitation assay (RIPA) lysis buffer containing 50 mM Trise HCl, 150 mM NaCl, 5 mM EDTA, 1% Triton X-100, 1 mM sodium fluoride, 1 mM sodium vanadate, 1% deoxycholate, and protease inhibitor cocktail. Lysates were cleared by centrifugation at 12,000× *g* for 10 min at 4 °C and the supernatants containing proteins were collected. SDS-PAGE electrophoresis was then performed, followed by protein transfer to a nitrocellulose membrane. The membranes were exposed to appropriate primary antibodies and then their corresponding secondary antibodies. The immunoreactivity was visualized using Enhanced Chemiluminescence (ECL) reagents (Amersham, Shanghai, China) according to manufacturer’s instructions. Three independent experiments were performed and the intensity of each band was analyzed using the Image J software.

### 4.16. Immunofluorescent Staining for NF-κB Localization 

MH7A cells were plated at a density of 1.5 × 10^4^/well in 24-well plates containing sterile cover slips and grown at 37 °C for 24 h. Following serum starvation, the cells were treated with JH (2.5 µM) for 4 h, and then stimulated with TNFa (50 ng∙mL^−1^) for 30 min. The cells were then washed twice with PBS and fixed onto the cover slips by methanol for 20 min. After washing with PBS, the cells were permeabilized with 0.5% Triton-X 100 for 10 min. Cover slips were blocked in 10% goat serum for 1 h at room temperature. NF-κB p65 antibody was added to the cover slips and incubated overnight at 4 °C. For nuclear staining, DAPI solution (Sigma-Aldrich, St. Louis, MI, USA) was added and incubated for 10 min in the dark. The nuclear translocation of p65 was imaged using a Nikon A1R resonance scanning confocal microscope with spectral detector (Nikon, Tokyo, Japan).

### 4.17. Statistical Analysis

All data were expressed as the mean ± SD of results obtained from three or more experiments. Statistical comparisons were performed using one-way ANOVA, followed by Tukey’s post hoc analysis. *p* < 0.05 was considered statistically significant.

## 5. Conclusions

In summary, we have demonstrated the therapeutic potential of JH on CIA rats in vivo and MH7A cells in vitro. The antiarthritic actions of JH might attribute to its inhibitory effect on NF-κB and MAPKs (ERK and p38) signaling pathways which further downregulated the production of proinflammatory mediators, and these decreased levels of cytokines might lead to the antiproliferative and antimigratory activities of rheumatoid FLS. Thus, JH has the potential to be researched and developed into a novel therapeutic natural agent for treatment of RA.

## Figures and Tables

**Table 1:** Effects of JH on the change of body weight, organ weight, and biochemical parameters in rats with collagen-induced arthritis.

Parameters	Control	Vehicle	MTX (3 mg∙kg^−1^)	JH (20 mg∙kg^−1^)	JH (50 mg∙kg^−1^)
Body weight gain (%)	12.38 ± 2.18	−1.05 ± 1.07 ^###^	0.08 ± 0.60	2.63 ± 0.69 *	3.5 ± 1.55 **
Liver (g)	8.69 ± 0.29	7.59 ± 0.77	7.67 ± 0.94	7.27 ± 0.59	7.51 ± 0.72
Spleen (g)	0.67 ± 0.055	0.57 ± 0.059	0.62 ± 0.061	0.63 ± 0.061	0.65 ± 0.98
AST (U/L)	22.47 ± 3.25	23.54 ± 7.77	24.66 ± 5.93	24.54 ± 3.61	22.41 ± 4.72
ALT (U/L)	4.56 ± 2.78	3.27 ± 1.87	2.90 ± 1.52	3.39 ± 2.88	3.24 ± 2.11

Rats were orally administrated with JH (20 and 50 mg·kg^−1^) or 0.9% saline daily for up to 14 days. The MTX-treated group received intragastrical administration with MTX (3 mg·kg^−1^) every 3 days as a positive agent. Data are expressed as mean ± SD; *n* = 6 in age-matched control group and *n* = 7 in vehicle and MTX- or JH-treated groups, respectively. ^###^ *p* < 0.001 versus control (age-matched rats); * *p* < 0.05 and ** *p* < 0.01 versus vehicle-treated CIA rats. ALT, alanine aminotransferase; AST, aspartate aminotransferase.
